# Polycarbonate as an Elasto-Plastic Material Model for Simulation of the Microstructure Hot Imprint Process

**DOI:** 10.3390/s130911229

**Published:** 2013-08-22

**Authors:** Birutė Narijauskaitė, Arvydas Palevičius, Rimvydas Gaidys, Giedrius Janušas, Rokas Šakalys

**Affiliations:** 1 International Studies Centre, Kaunas University of Technology, A. Mickevičiaus Gatvė 37, Kaunas 44244, Lithuania; E-Mails: birute.narijauskaite@ktu.lt (B.N.); arvydas.palevicius@ktu.lt (A.P.); rokas.sakalys@ktu.lt (R.Š.); 2 Department of Engineering Design, Kaunas University of Technology, Kęstučio Gatvė 27, Kaunas 44025, Lithuania; E-Mail: rimvydas.gaidys@ktu.lt

**Keywords:** elasto-plastic, hot imprint, finite element method, polycarbonate

## Abstract

The thermal imprint process of polymer micro-patterning is widely applied in areas such as manufacturing of optical parts, solar energy, bio-mechanical devices and chemical chips. Polycarbonate (PC), as an amorphous polymer, is often used in thermoforming processes because of its good replication characteristics. In order to obtain replicas of the best quality, the imprint parameters (e.g., pressure, temperature, time, *etc.*) must be determined. Therefore finite element model of the hot imprint process of lamellar periodical microstructure into PC has been created using COMSOL Multiphysics. The mathematical model of the hot imprint process includes three steps: heating, imprinting and demolding. The material properties of amorphous PC strongly depend on the imprint temperature and loading pressure. Polycarbonate was modelled as an elasto-plastic material, since it was analyzed below the glass transition temperature. The hot imprint model was solved using the heat transfer and the solid stress-strain application modes with thermal contact problem between the mold and polycarbonate. It was used for the evaluation of temperature and stress distributions in the polycarbonate during the hot imprint process. The quality of the replica, by means of lands filling ratio, was determined as well.

## 1. Introduction

Nowadays microstructures have a very wide range of applications. They are used for beam shaping, splitting and steering [1], in optical interconnections [2], optical tweezers [3], multiphoton spectroscopy [4], lithographic fabrication of photonic crystals [5], object contouring [6], biological microscopy [7], measurement of moving object [8], characterization of micro optical elements, systems with CD and DVD [9], in X-ray microscopy [10], *etc.* Therefore the quality of produced microstructures is very important. E-beam lithography (EBL) is the most commonly used technique for micro- or nanolithography. The usage of EBL for pattering offers many advantages, which provide an ideal lithographic platform for the MEMS (NEMS) fabrication. On the other hand, this technology is better suited for prototyping, but not for mass production [11]. In mass production well-known conventional technologies, like injection molding, injection compression molding and hot embossing are used. They are extensively employed in micro scale replication.

Hot embossing is the most popular manufacturing process, and is well suited for producing dedicated microstructures with high aspect ratios and small distortions [12,13]. Hot embossing has several advantages over other replication processes, including relatively low costs for embossing tools, a simple process, and high replication accuracy for small features. However many scientists highlight the following hot imprint process problems:

- filling ratio of microstructure [14];
- non-uniform mold imprint [15];
- adhesion between mold and polymer [16];
- surface roughness [17];
- long cycle time [18].

However, it is not enough to optimize the parameters of hot imprint process. It is also necessary to take into account the different technological equipment, materials, *etc.* This requires new methods and areas for improvement in order to achieve better quality of replicas. The reasons for these problems can be detected using numerical simulation.

The literature analysis shows that there is not enough information about polycarbonate material behavior in mechanical hot imprint processes. The imprint process involves finite temperature-dependent deformation of a thermoplastic material. As a result, it is costly to establish experimentally the process behavior for these materials, leading to a critical need for improved simulation capabilities. The material behavior is highly sensitive to variations of temperature and strain rate. Furthermore, numerical simulation is essential to push the limits of hot imprinting to smaller length scales where high precision is critical. The polymethyl methacrylate (PMMA) is most numerically analyzed at different temperatures and state of the material. Therefore, an elastoplastic polycarbonate material model is designed and analyzed by the finite element method in this paper. It is necessary to investigate the stress and strain state in each step of the process. The filling ratio of the polymer in the mechanical hot imprint process is studied based on numerical simulation and experimental research. Heating, imprinting, and demolding steps in the hot imprint process are investigated in detail by a numerical simulation study. Therefore the aim of this paper is to create finite element model (FEM) of a nickel mold hot imprint into polycarbonate, which corresponds to the experiment conditions.

## 2. Finite Element Model of Mechanical Hot Imprint Process

**Figure 1.**
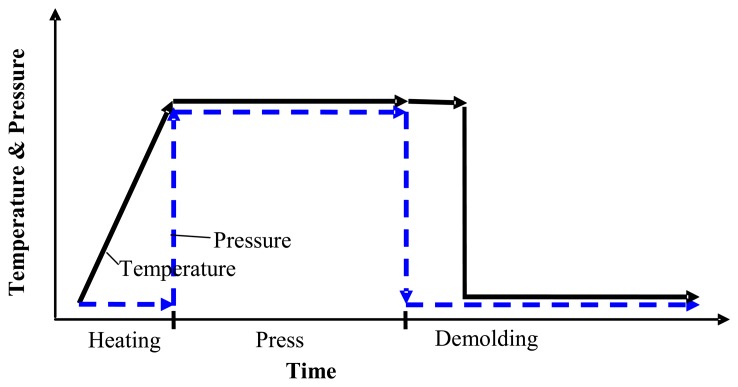
Diagram of the mechanical hot imprint process.

The finite element model of the nickel mold hot imprint process into polycarbonate near the glass transition temperature, which is created with COMSOL Multiphysics 3.5a, is presented in this section. Many scientists divide the mechanical hot imprint process into four steps: heating, imprint, cooling and demolding. However we are making assumption that after the imprint step the temperature of polymer decreases very quickly. Therefore the cooling step was not analyzed separately. The scheme of the modified hot imprint process, which consists of three steps: heating, imprinting and demolding, is presented in Figure 1.

**Figure 2.**
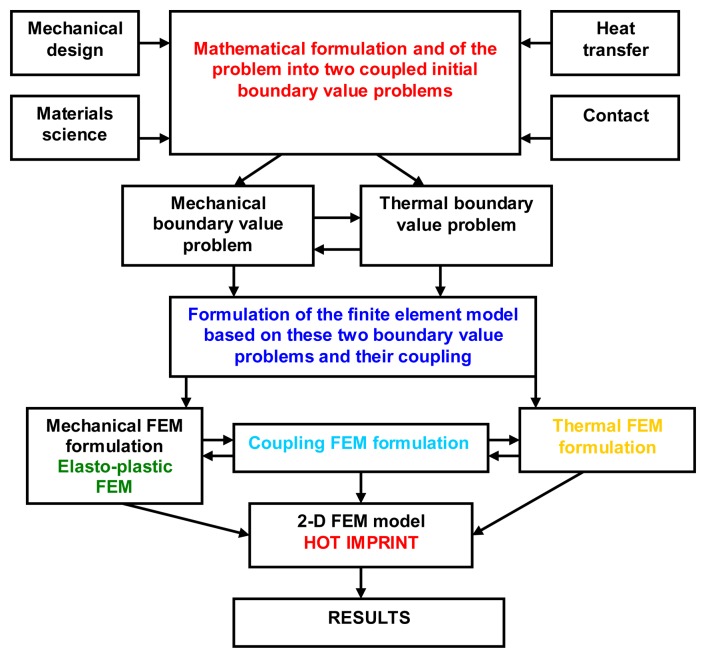
The hot imprint process modeling.

The modeling and simulation methodology by FEM, which includes geometrical modeling, boundary conditions, meshing, material properties, process conditions and governing equations is schematically presented in Figure 2. The equations of motion, thermal balance, material properties and material deformation were used in order to calculate the stress, strain, and temperature fields, the mold pressure distribution and filling ratio in each step of hot imprint process.

The Green-Lagrange strain-displacement Equation (1) describes the basic relations between large displacements and strain:

(1) $$\varepsilon =\left\{\begin{array}{c} \varepsilon_{x}\\ \varepsilon_{y}\\ \varepsilon_{z}\\ \gamma_{yz}\\ \gamma_{zx}\\ \gamma_{xy} \end{array}\right\}=\left\{\begin{array}{c} \frac{\partial u}{\partial x}+\frac{1}{2}\left(\frac{\partial^{2}u}{\partial x^{2}}+\frac{\partial^{2}v}{\partial x^{2}}+\frac{\partial^{2}w}{\partial x^{2}}\right)\\ \frac{\partial u}{\partial y}+\frac{1}{2}\left(\frac{\partial^{2}u}{\partial y^{2}}+\frac{\partial^{2}v}{\partial y^{2}}+\frac{\partial^{2}w}{\partial y^{2}}\right)\\ \frac{\partial u}{\partial z}+\frac{1}{2}\left(\frac{\partial^{2}u}{\partial z^{2}}+\frac{\partial^{2}v}{\partial z^{2}}+\frac{\partial^{2}w}{\partial z^{2}}\right)\\ \frac{\partial v}{\partial z}+\frac{\partial w}{\partial y}+\left(\frac{\partial u}{\partial y}\frac{\partial u}{\partial z}+\frac{\partial v}{\partial y}\frac{\partial v}{\partial z}+\frac{\partial w}{\partial y}\frac{\partial w}{\partial z}\right)\\ \frac{\partial w}{\partial x}+\frac{\partial u}{\partial z}+\left(\frac{\partial u}{\partial z}\frac{\partial u}{\partial x}+\frac{\partial v}{\partial z}\frac{\partial v}{\partial x}+\frac{\partial w}{\partial z}\frac{\partial w}{\partial x}\right)\\ \frac{\partial u}{\partial y}+\frac{\partial v}{\partial x}+\left(\frac{\partial u}{\partial x}\frac{\partial u}{\partial y}+\frac{\partial v}{\partial x}\frac{\partial v}{\partial y}+\frac{\partial w}{\partial x}\frac{\partial w}{\partial y}\right) \end{array}\right\}$$

Here *ε_x_*, *ε_y_*, *ε_z_*, *γ_yz_*, *γ_xz_*, *γ_xy_* are linear strains in the *x*, *y*, *z* direction and shear strains in the *yz*, *xz*, *xy* plane, while u, *v*, *w* are displacements in the *x*, *y*, *z* direction.

Heat transfer conductivity is described according to the formula:

(2) $$\rho (T)c_{p}(T)\frac{\partial T}{\partial t}+\nabla \left(-k\nabla T\right)=q$$

where *k* is thermal conductivity, *ρ* is density, *c_p_* is heat capacity, *T* is temperature, *q* is rate of the heat generation.

COMSOL Multiphysics solves contact problems using an augmented Lagrangian method. This method is a combination of the penalty and Lagrange multiplier methods. This means a penalty method with penetration control. The system is solved by iteration from the determined displacement. These displacements caused by incremental loading, are stored and used to deform the structure to its current geometry. If the gap distance between the slave and master boundaries at a given equilibrium iteration is becoming negative (*i.e.*, the master boundary is penetrating the slave boundary), the user defined normal penalty factor *p_n_* is augmented with Lagrange multipliers for contact pressure *T_n_*:

(3) $$T_{np}=\left\{ \begin{array}{ll} T_{n}-p_{n}g & if\;g\leq 0\\ T_{n}e^{-\frac{p_{n}g}{T_{n}}} & \text{otherwise} \end{array}\right.$$

where *g* is the gap distance variable between slave and master boundary [19,20].

**Figure 3.**
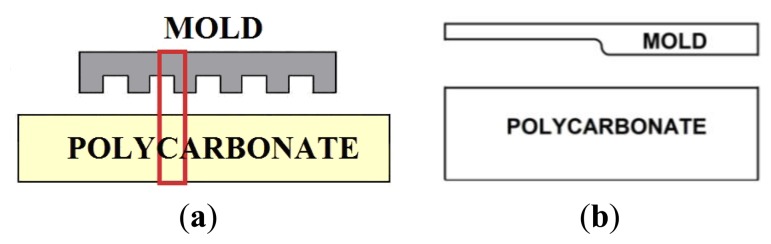
Cross-sectional shape of (**a**) the mold and substrate, and (**b**) the analyzed section.

Generally it is impractical to use FEM to analyze periodical micrometer-scale patterning of the mold, but if the cross-sectional shape of the mold is constant in one direction, as in Figure 3, two-dimensional stress analysis is possible. Moreover, if the pattern of the mold is regular and symmetric, we can assume a two-dimensional plane strain model of a unit cell (Figure 3) by taking symmetric boundary conditions into account.

**Figure 4.**
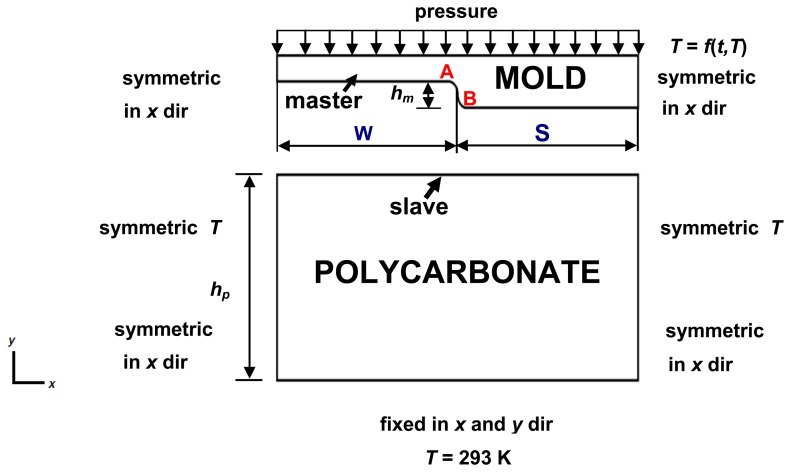
Computational scheme of the nickel mold hot imprint into polycarbonate.

A two-dimensional (2-D) FEM model of a nickel mold (lamellar profile with period of 4 μm) and polycarbonate substrate with boundary conditions is presented in Figure 4, where depth of the mold (*h_m_*) is 100 nm, thickness of the polycarbonate (*h_p_*)—3 mm, half width of the land (*W*)—1 μm, half width of the ridge (*S*)—1 μm. The mold is made of nickel alloy, which is a more rigid material than polycarbonate, so it was assumed in the FE simulation that the mold has rigid contact surfaces. Symmetric boundaries were used on the left and right sides of the model. A symmetric boundary indicates that displacement and temperature gradients across the boundary are zero. Fixed displacement and temperature are applied at the bottom surface of the substrate. The initial temperature of the mold and polycarbonate is 293 K. During the preheating step the mold's temperature was defined as linear function *T* = *f*(*T*, *t*) of mold's heating temperature *T* (Kelvin) and mold displacements *t* (meters). When the temperature reaches the maximum value (421 K), it remains stable during all further steps. The air influence between mold and polycarbonate is neglected. Also in order to improve the convergence of the simulation and avoid stress concentrations small radius arcs were implemented in the A and B areas of the mold (Figure 4). The pressure is described as a linear function *f*(*t*) dependent on mold displacement *t* from 0 to 9 × 10^−7^ m with 10^−8^ m steps.

**Figure 5.**
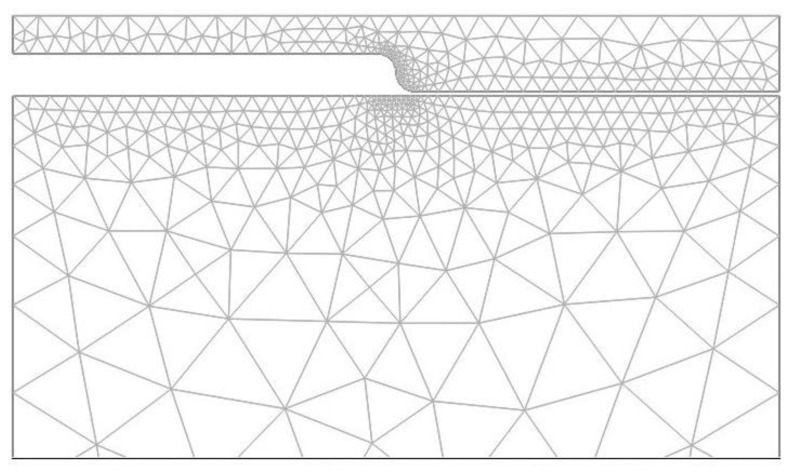
Finite element mesh of the system of nickel mold and polycarbonate.

The accuracy and convergence of the solution depends on the choice of mesh as well. The mesh of the model, using triangular elements, is presented in Figure 5. The triangular element is defined by six nodes, each having three degrees of freedom: horizontal *x* and vertical *y* displacement, and temperature. The Lagrange-Quadratic finite element type, which is used for 2-D modeling of solid structures, was chosen. Meshes of the contact area and at the symmetrical regions are finer than in other areas with smaller deformation. The model consists of 5,583 finite elements. It is recognized that interaction between structural parts has a great influence on the results for solving a multi-field contact problem. Smoothing of contact edges provides a significant improvement in convergence behavior and the refiner mesh allows one to define contact accurately. The refined mesh was used around arcs so that more points come onto contact at the same time. The arc of the mold's contact edge consists of 12 nodal points. The horizontal molding and PC contact edges were discretised coarsely (mesh 3 times spare) and have the same mesh density. Comparisons of numerical and theoretical results show that the defined mesh ensures the necessary accuracy of the solution.

Materials used for the mold and substrate, and their properties are listed in Table 1. Nickel was used as the mold, and it was assumed to be isotropic and linearly elastic, while amorphous polycarbonate (glass transition temperature 423 K) was used as substrate.

**Figure 6.**
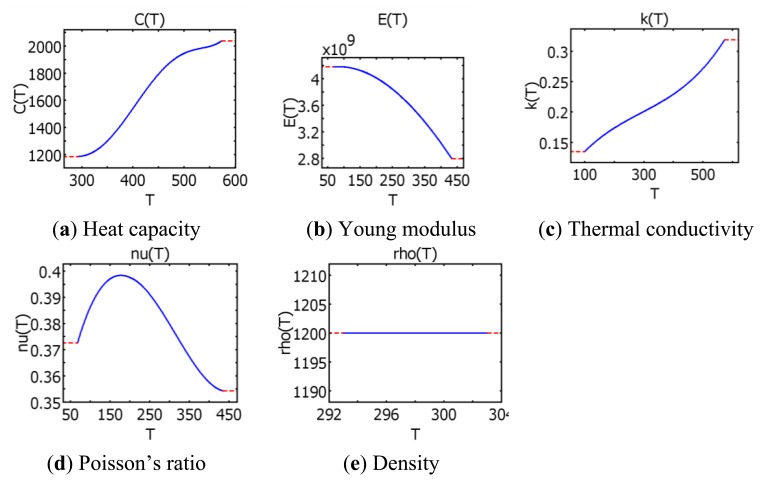
Thermal dependencies of polycarbonate material properties (COMSOL Multiphysics 3.5a material library).

Polycarbonate's density, thermal conductivity, heat capacity, elastic modulus and Poisson's ratio as functions of temperature were taken from COMSOL Multiphysics 3.5a material library (Figure 6).

In the polycarbonate during hot imprint process large deformations are induced. The micro hot imprint process is being performed near the glass transition temperature of polycarbonate, where it behaves like elasto-plastic material. The mold and substrate are restrained from moving in the *z* direction. Therefore it is possible to use the 2D Plane Strain application mode, which assumes, that the *z*-component of the strain is zero. The total strain vector *ε* of polycarbonate material consists of thermal *ε_th_* and elastic *ε_el_* strain vectors so that:

(4) $$\varepsilon =e_{el}+\varepsilon_{th}$$

The polycarbonate material model with a nonlinear behavior is an elasto-plastic material, where the stress-strain relationship or the constitutive equation is:

(5) $$\sigma =D\varepsilon_{el}=D\left(\varepsilon -\varepsilon_{p}-\varepsilon_{th}\right)$$

where *D* is the elasticity matrix and the stress and the strain are both given in column vector form:

(6) $$\begin{array}{ll} \sigma =\left[\begin{array}{c} \sigma_{x}\\ \sigma_{y}\\ \sigma_{z}\\ \tau_{xy}\\ \tau_{yz}\\ \tau_{xz} \end{array}\right], & \varepsilon =\left[\begin{array}{c} \varepsilon_{x}\\ \varepsilon_{y}\\ \varepsilon_{z}\\ \gamma_{xy}\\ \gamma_{yz}\\ \gamma_{xz} \end{array}\right] \end{array}$$

Thermal strain depends on the present temperature *T*, the stress-free reference temperature *T*_ref_, and the thermal expansion vector α_vec_:

(7) $$\varepsilon_{th}={\left[\begin{array}{c} \varepsilon_{x}\\ \varepsilon_{y}\\ \varepsilon_{z}\\ \gamma_{xy}\\ \gamma_{yz}\\ \gamma_{xz} \end{array}\right]}_{th}=\alpha_{\text{vec}}\left(T-T_{\text{ref}}\right)$$

For elasto-plastic material:

(8) $$\alpha_{\text{vec}}=\left[\begin{array}{c} \alpha \\ \alpha \\ \alpha \\ 0\\ 0\\ 0 \end{array}\right]$$

The polycarbonate as an elasto-plastic material that has yield criterion and hardening model settings. The yield criterion is interpreted as an equivalent stress *σ_e_*. When the equivalent stress is equal to a material yield parameter *σ_Y_* the material will develop plastic strains. If *σ_e_* is less than *σ_Y_*, the material is elastic and the stresses will develop according to the elastic stress-strain relations. Equivalent stress can never exceed the material yield since in this case plastic strains would develop instantaneously, thereby reducing the stress to the material yield. As a yield function the Von Mises function was chosen:

(9) $$\sigma_{Y}=\sqrt{\frac{1}{2}{\left(\sigma_{1}-\sigma_{2}\right)}^{2}+{\left(\sigma_{2}-\sigma_{3}\right)}^{2}+{\left(\sigma_{1}-\sigma_{3}\right)}^{2}}$$

where *σ*_1_, *σ*_2_, *σ*_3_ are principal stresses and *σ_Y_* is the equivalent stress [21].

The yield stress level is given by:

(10) $$\sigma_{Y}(T)=E\left(T\right)\cdot \varepsilon_{y}\left(T\right)$$

where *T*—present temperature, *E*(*T*)—Young's modulus function, *ε_y_(T)*—yield strain function.

The polycarbonate *E*(*T*) function is chosen from the COMSOL Multiphysics material library, *ε_y_(T)* function was created as a linear function according to results published in [22,23]. Yield strain varies from 8% to 5%, when the temperature varies from 293 K to 417 K.

The hardening model is a phenomenon where yield stress increases with further plastic strain. Isotropic hardening has been proposed to define the modification of the yield surface during plastic deformation. Using this hardening model, it was assumed that the initial yield surface expands uniformly without distortion and translation as plastic flow occurs. The isotropic tangent modulus *E_Tiso_* = *MPa* [22,23]. A Lagrangian formulation was used for incremental general nonlinear analysis in COMSOL Multiphysics.

The model was solved using heat transfer and the solid stress-strain application modes with thermal contact problem between nickel mold and polycarbonate. This multiphysical hot imprint model of polycarbonate includes the heat transport, structural mechanical stresses and strains resulting from the temperature distribution. It allows us to evaluate temperature distributions and stresses in the polycarbonate during the hot imprint process.

## 3. Results and Discussion

**Figure 7.**
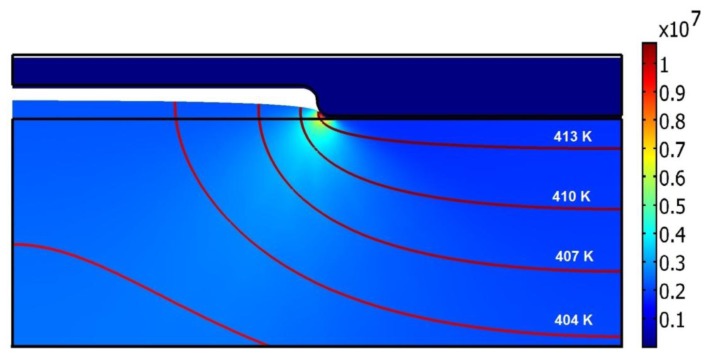
Von Mises stress distribution (color map, Pa) and temperature fields (in Kelvins) represented by lines in the polycarbonate after the heating process.

**Figure 8.**
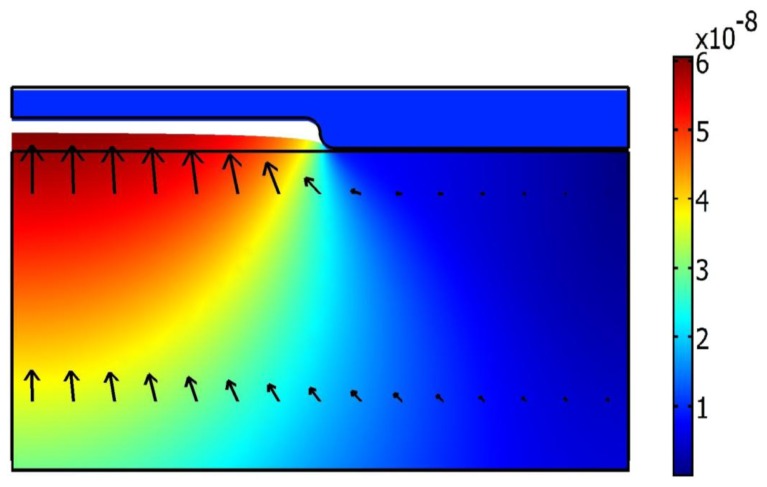
Distribution of total displacements (in meters) after heating process.

As described in the previous section, the hot imprint process was divided into three steps: heating, imprinting and demolding. The initial temperature of the mold and polycarbonate is 293 K, the same as the ambient temperature and the imprint force is equal to zero at the beginning of the heating step. In this step the temperature of the mold increases up to the chosen 421 K and through the initial contact between mold ridges and polycarbonate substrate it was preheated (Figure 7). During the heating process, deformation of polycarbonate starts (Figure 8).

Polycarbonate is elastic, due to this the polymer from the contact area moves to the empty cavity of the mold. The cavity of the mold is partially filled with heated polycarbonate. After the heating a steady variation of temperature in the range from 295 K (in the bottom of polycarbonate) to 413 K (in the place of contact with the mold) was observed; this is presented by contour lines (Figure 7). Von Mises stress in polycarbonate reaches 10.6 MPa (Figure 7).

**Figure 9.**
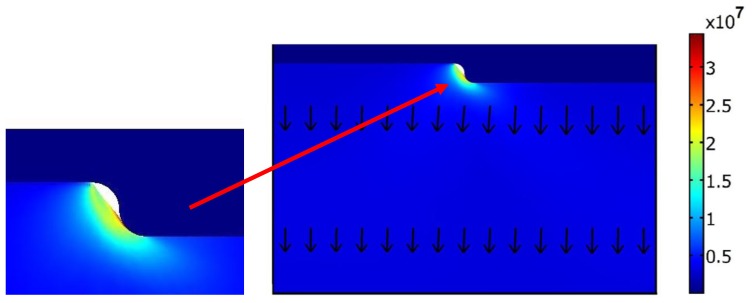
Von Mises stress distribution in the deformed polycarbonate (in Pa) and total displacements (in meter) represented by arrows after imprint step.

During the imprint step (Figure 9), the mold moves down from the initial point about 900 nm (it corresponds 5 atm pressure) and presses the polycarbonate, at the same time the contact force between the mold and polycarbonate increases and plastic strains appears. Maximum Von Mises stress (33.6 MPa) is located in the contact area between the mold's corner and polycarbonate. The arrows in Figure 9 show that total displacement after the hot imprint process is diverted downwards.

**Figure 10.**
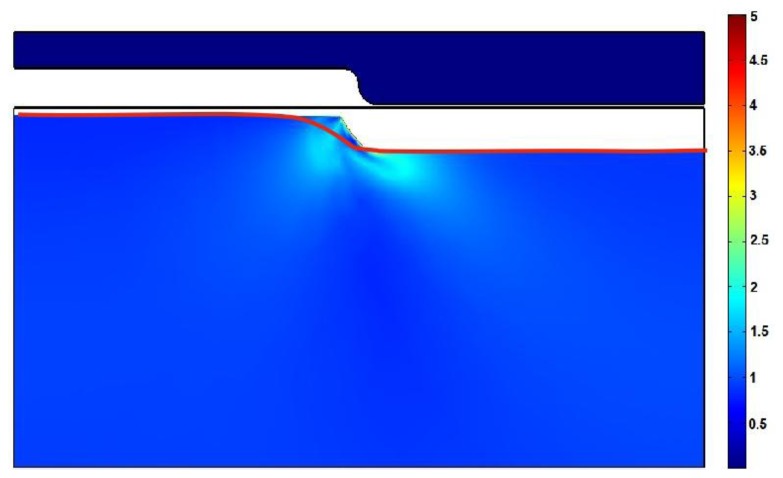
Areas of the permissible yield and profile of the experimental microrelief represented by the red line.

In the demolding step, the hot mold (*T* = 421 K) is demolded and finally polycarbonate is cooled, and it sustains the form of the mold (Figure 10).

**Figure 11.**
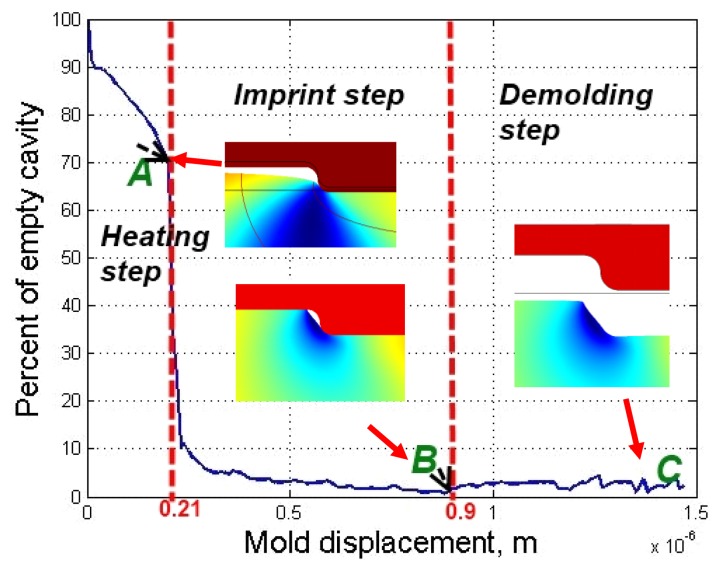
The dependence of non-filling cavity *versus* mold displacement.

One of the most important qualitative parameters in a hot imprint process is the filling ratio of the mold's microrelief. It is defined as a ratio of filled area and total area of microstructure. Figure 10 represents dependence of the non-filled cavity on mold displacement through the whole hot imprint process. As shown in Figure 11, non-filled cavity decreases very slowly during the heating step. At the end of this step the filling ratio is about 70%, and the mold's displacement 2 × 10^−7^ m. Then the empty cavity decreases very quickly (about seven times) to 10%, because the top surface of the polycarbonate becomes a soft material (it reaches the glass transition temperature). Through the remaining part of the imprint step, the empty cavity decreases to 2%. After the demolding step the empty cavity increases slightly and remains at about 4%.

The elastic strain appears when the mold displacement reaches 0.21 μm, and plastic strain appears when the mold displacement reaches 0.9 μm and then when the mold is demolded residual strains appear in the polycarbonate.

**Figure 12.**
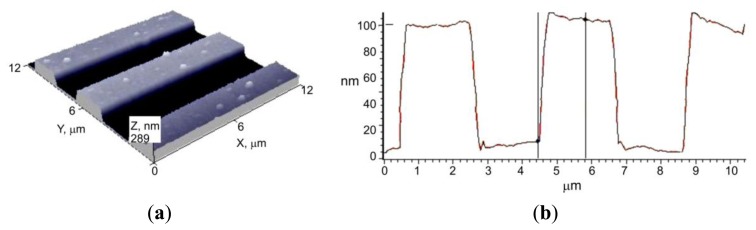
(**a**) 3D and (**b**) 2D view of the replica.

The numerical model was verified experimentally. The same experimental scheme as in the numerical simulation was used. A lamellar microstructure of 4 μm period and 100 nm depth was replicated into polycarbonate (3 mm thickness) for 15 seconds at 148 °C and under 5 atm pressure. Flat embossing experiment was performed using a flat thermal pressure device (designed at the Institute of Materials Science of Kaunas University of Technology, Kaunas, Lithuania). The original construction ensures controlled pressure, force, temperature and exposure time (*P* = 1–5 atm, *T* = 20–200 °C, *t* = 1–300 s). During the experiment, the mold was being heated up to the required temperature, before the microstructure was imprinted onto the polymer. During the mechanical hot imprint process, the polymer was compressed with a high load. The polymer is plastic, so after demolding it remains deformed. A view of the replica, obtained by using atomic force microscopy, is presented in Figure 12.

In order to compare the area of the microstructure imprinted onto the polycarbonate with the area of the nickel mold, data from AFM measurements were used. Experimental results were integrated using Simpson's rule. Calculations show that the area not filled with polycarbonate is about 10%, whereas the theoretically empty area is 4%. Graphical comparison of the theoretical microrelief with the experimental one (red line) is presented in Figure 10. This shows that the difference between model and reality is not big and model is suitable for theoretical evaluation of the material behavior during the thermal imprint process.

## 4. Conclusions

A mathematical non-linear model of the hot imprint process of a nickel mold into polycarbonate was created using an elasto-plastic material model. The finite element model, implemented by COMSOL Multiphysics software, allows us to determine temperature fields, displacements and stresses in each step of the hot imprint process. The filling ratio of the mold is the main parameter of the replica's quality. Therefore dependence of the empty cavity *versus* the mold's imprint displacement was obtained in all steps of the hot imprint process. Numerically it was determined that after the hot imprint process the empty cavity remains at about 4%.

The finite element model was verified using experimental investigations. In the experimental and numerical results, lamellar form replicas were observed, and differences between experimentally and numerically obtained filling ratios are within allowable limits. In addition the average measured depth of the replica is about 100 nm, the same as the calculated value.

## Figures and Tables

**Table 1.** Material properties of the mold and substrate.

	**Mold**	**Substrate**
**Material**	Nickel	Polycarbonate
**Density, Kg/m^3^**	8.908 × 10^3^	*ρ*(*T*)
**Thermal conductivity, W/mK**	90.9	*K*(*T*)
**Thermal expansion, 1/K**	13.4 × 10^−6^	6.5 × 10^−5^
**Heat capacity, J/(Kg·K)**	445	*c_p_*(*T*)
**Elastic modulus, GPa**	200	*E*(*T*)
**Poisson's ratio**	0.31	*ν* (*T*)
